# Towards a universal vaccine for avian influenza: Protective efficacy of modified Vaccinia virus Ankara and Adenovirus vaccines expressing conserved influenza antigens in chickens challenged with low pathogenic avian influenza virus

**DOI:** 10.1016/j.vaccine.2012.11.047

**Published:** 2013-01-11

**Authors:** Amy C. Boyd, Raul Ruiz-Hernandez, Marylene Y. Peroval, Connor Carson, Devanand Balkissoon, Karen Staines, Alison V. Turner, Adrian V.S. Hill, Sarah C. Gilbert, Colin Butter

**Affiliations:** a The Jenner Institute, Oxford University, Oxford, UK; b The Pirbright Institute, Compton Laboratory, Compton, UK

**Keywords:** AI, avian influenza, NP + M1, fusion construct of influenza nucleoprotein and matrix protein, SFU, spot-forming units, dpb, days post boost, dpi, days post infection, wph, weeks post hatch, SPF, specific pathogen free, Influenza, LPAI, Vaccine, Chicken, MVA, Adenovirus

## Abstract

Current vaccines targeting surface proteins can drive antigenic variation resulting either in the emergence of more highly pathogenic viruses or of antigenically distinct viruses that escape control by vaccination and thereby persist in the host population. Influenza vaccines typically target the highly mutable surface proteins and do not provide protection against heterologous challenge. Vaccines which induce immune responses against conserved influenza epitopes may confer protection against heterologous challenge. We report here the results of vaccination with recombinant modified Vaccinia virus Ankara (MVA) and Adenovirus (Ad) expressing a fusion construct of nucleoprotein and matrix protein (NP + M1). Prime and boost vaccination regimes were trialled in different ages of chicken and were found to be safe and immunogenic. Interferon-γ (IFN-γ) ELISpot was used to assess the cellular immune response post secondary vaccination. *In ovo* Ad prime followed by a 4 week post hatch MVA boost was identified as the most immunogenic regime in one outbred and two inbred lines of chicken. Following vaccination, one inbred line (C15I) was challenged with low pathogenic avian influenza (LPAI) H7N7 (A/Turkey/England/1977). Birds receiving a primary vaccination with Ad-NP + M1 and a secondary vaccination with MVA-NP + M1 exhibited reduced cloacal shedding as measured by plaque assay at 7 days post infection compared with birds vaccinated with recombinant viruses containing irrelevant antigen. This preliminary indication of efficacy demonstrates proof of concept in birds; induction of T cell responses in chickens by viral vectors containing internal influenza antigens may be a productive strategy for the development of vaccines to induce heterologous protection against influenza in poultry.

## Highlights

► Current influenza vaccines do not generate heterologous protection. ► Targeting internal influenza antigens may confer cross protection. ► We tested Adenovirus and MVA vectored NP and M1 in chickens. ► Heterologous prime-boost resulted in earlier cessation of viral shedding.

## 1 Introduction

As zoonotic pathogens with pandemic potential, avian influenza (AI) viruses represent a significant threat to human health. Outbreaks of AI in domestic birds also place a significant financial burden on the global poultry industry, threatening food security by raising food prices in the face of increasing world protein demand. Commercial vaccines targeting AI surface proteins such as haemagglutinin offer limited protection against heterologous viral strains. As vaccination for AI has become more common, the level of antigenic drift in avian viruses has increased. In Mexico, long term vaccination coupled with a failure to eradicate low pathogenic H5N2 AI has resulted in the observation that similar levels of antigenic drift can now be seen in avian viruses compared with human viruses [1]. Vaccination without eradication increases the likelihood that antigenically distinct and potentially more virulent strains of AI will arise and a major goal of vaccination is therefore to produce sterilising immunity. An ideal AI vaccine would offer heterologous protection and eliminate shedding of infectious virus. Current vaccination strategies induce mainly humoral immunity which is not sustainably cross protective as viruses diversify away from vaccine specificity. A more effective approach aims to include the induction of T cell responses which have been shown to confer protection against heterologous influenza virus strains in humans and mice [2,3]. Recent research has shown that in human volunteers vaccination with MVA-NP + M1 successfully boosted pre-existing cellular immune responses to seasonal influenza, resulting in significantly increased *ex vivo* T cell IFN-γ ELISpot responses to NP and M1 peptides [4] and significant reduction in duration of viral shedding in vaccinated volunteers following influenza virus challenge [5].

Cross reactive cell mediated responses are thought to play a similar role in the chicken although there is a paucity of literature [6]. These responses target conserved epitopes of internal antigens which are subject to lesser degrees of antigenic drift compared to antigens of the surface proteins haemagglutinin and neuraminidase. A significant benefit of a vaccine targeting conserved internal antigens is the cell mediated destruction of viral factories and hence reduction in viral load and shedding, irrespective of the variation of surface antigens. Conserved internal proteins thought to contribute to cross protection include nucleoprotein and matrix protein. Recent studies have demonstrated the utility of nucleoprotein in inducing T cell responses in chickens, although these did not investigate protection against challenge with influenza virus [7,8]. To investigate cross protective responses in chickens we have established prime and boost vaccination regimes with recombinant modified Vaccinia virus Ankara (MVA) and Adenovirus (Ad) expressing a fusion construct of nucleoprotein and matrix protein (NP + M1). M1 is a known target for T cells [3] and as a conserved internal antigen was expected to confer protection in the same way. Additionally the inclusion of another antigen increases the number of potential protective epitopes. The objectives of this study were to establish the safety and immunogenicity of the NP + M1 construct in viral vector delivery systems in domestic poultry, to investigate optimisation of regimes and to determine vaccine efficacy against challenge with low pathogenic avian influenza (LPAI).

## 2 Materials and methods

### 2.1 Experimental animals

Specific pathogen free (SPF) inbred white leghorns (Line 6_1_ and 15I) and outbred Light Sussex chickens were supplied as 17–19 days embryos by the Poultry Production Unit of The Pirbright Institute, Compton laboratory. Birds were hatched and maintained in SPF containment with *ad libitum* access to water and commercial chicken feed. For influenza challenge birds were transferred to Bioflex B50 Rigid Body Poultry isolators (Bell Isolation Systems Ltd, UK) and allowed to acclimatise prior to infection. Birds were examined daily for clinical signs of disease. Experimental procedures were carried out in accordance with Ethical Review and UK Home Office requirements.

### 2.2 Vaccination regimes

MVA and non-replicating human Adenovirus 5 (Ad) vectors expressing a fusion of nucleoprotein (NP) and matrix protein (M1) from influenza A/Panama/2007/99 (H3N2) and Ad and MVA-vectored constructs containing GFP as irrelevant antigen controls were supplied by the Vector Core Facility at the Jenner Institute (Oxford, UK). The NP + M1 antigen comprises the complete sequences of NP and M1 from A/Panama/2007/99 fused by a 7 amino acid linker sequence. Expression of the fusion protein in MVA is controlled by the Vaccinia P7.5 promoter inserted at the thymidine kinase locus. Recombinant MVA was generated in primary chick embryo fibroblast (CEF) cells. In Ad, which is E1 and E3 deleted, the human CMV promoter is used to drive expression of the fusion protein. Recombinant Ad was generated in 293 cells. These are replication-deficient viral vectors that do not persist *in vivo* following immunisation, and it is expected that antigen expression lasts from a few hours (MVA) to a few days (Ad5). Priming vaccination was performed *in ovo* at 17–19 days of incubation. 1 × 10^9^ IU (infectious units) Ad-NP + M1 or Ad-GFP in 100 μL of sterile PBS was injected into the amniotic cavity with a 23 gauge needle to an approximate depth of 1 in. Boost vaccination with 1 × 10^7^ pfu (plaque forming units) MVA-NP + M1 or MVA-GFP in 100 μL of sterile PBS was administered by intramuscular (IM) injection (see appropriate results section text for precise regime details). Quantification of Ad was performed by infecting cell lines and then staining for foci of infection. This quantifies infectious virus particles per volume. IU for adenovirus is stated rather than PFU as we are assaying the ability of the virus particles to infect a single cell rather than to form a plaque resulting from the initial infection of a single cell followed by spreading of the infection to surrounding cells.

### 2.3 Sampling/swabbing

Swabbing of the buccal and cloacal cavities of experimental birds was carried out every 2–3 days for 2 weeks post infection with sterile polyester tipped swabs (Fisher Scientific, UK). Swab tips were transferred to 1 ml viral transport medium containing antibiotics and antimycotics, vortexed briefly and medium was either frozen at −80 °C for subsequent extraction of RNA or used within 2 days for plaque assays [9].

### 2.4 ELISpot

Post mortem spleens were macerated in sterile PBS supplemented with 1% foetal calf serum (Invitrogen, UK) and passed through a 100 μm cell strainer (Fisher, UK) before under-laying Histopaque 1119 (Sigma, UK) and centrifugation at 400 × *g* for 40 min. Cells at the interface were collected, washed twice in sterile PBS/1% FCS and resuspended in complete medium (RPMI with 2 mM Glutamax-I, supplemented with 10% foetal calf serum, 100 U/ml penicillin, and 100 mg/ml streptomycin (all Invitrogen, UK)). Cells were used immediately in ELISpot assays. Overlapping pools of 15–20-mer peptides covering the NP + M1 fusion construct sequence were used to measure T cell responses to vaccine antigens (Peptide Protein Research, Cambridge, UK). Pools of irrelevant peptides were used to control for background responses. IFN-γ ELISpot was carried out using the Chicken IFN-γ CytoSet™ (Life Technologies, UK) as described previously [10]. Briefly, 96-well multiscreen plates with Protein Binding Immobilon-P-Membrane (Millipore, Billerica,USA) were coated with 100 μL/well of 5 mg/ml mouse anti-chicken IFN-γ (Life Technologies, UK) in coating buffer (Sigma–Aldrich, UK) overnight at 4 °C. Plates were washed twice with blocking buffer (phosphate buffered saline supplemented with 2% FCS), then blocked with complete medium (see above) for 1 h at 37 °C. The blocking medium was discarded and 5 × 10^5^ splenocytes per well were incubated overnight at 41 °C in 5% CO_2_ in air, in the presence of non-specific mitogen (1 μg/ml phorbol 12-myristate 13-acetate (PMA, Sigma, UK) plus 1 μg/ml ionomycin (Sigma, UK)), recombinant NP protein (1:10 dilution of supernatant from S2 insect cells transfected with a plasmid expressing NP), beta-propiolactone inactivated LPAI (A/Turkey/England/1977/H7N7, 1 × 10^5^ pfu/ml) or NP + M1 peptide pools (10 μg/ml) in complete medium.

### 2.5 LPAI virus and challenge infection

LPAI virus (A/Turkey/England/1977/H7N7) was cultured in embryonated eggs using standard methods described elsewhere [9]. Viral titre was estimated by plaque assay on Madin-Darby canine kidney (MDCK) cells, using standard techniques [11]. Challenge infection was carried out 14 days after boost vaccination, by intra-nasal inoculation of LPAI (A/Turkey/England/1977 H7N7) at a dose of 3.4 × 10^7^ pfu in 100 μl of PBS per bird. The vaccine transgene and challenge virus NP sequences were 90.4% identical at the amino acid level. We do not have sequence data for the challenge virus M1, however alignments of a selection of representative M1 sequences suggest that >90% identity would be expected.

### 2.6 Plaque assay

Serial dilutions of viral transport medium from buccal and cloacal swabs were diluted in PBS then added to MDCK cells in duplicate wells in 6 well plates as described [11]. Final overlay was performed with 1× low gelling temperature agarose (Sigma, UK) in serum free culture medium containing TPCK trypsin (Sigma, UK) at 1.5 μg/ml. Cells were cultured at 37 °C for 72 h then stained with toluidine blue dye and plaques were counted.

### 2.7 RNA extraction and real time reverse transcriptase PCR

RNA was extracted from 275 μl viral transport medium using a commercial kit (QIAamp One-For-All Nucleic Acid kit (Qiagen, UK)) on an automated extraction system (Qiagen BioRobot Universal system (Qiagen, UK)). Primers and a probe specific for a conserved region of the Influenza A Matrix gene were used as described previously [12]. Single step real-time reverse transcriptase PCR was carried out using the Superscript III Platinum One-Step qRT-PCR Kit (Invitrogen, UK). Primers and probe were used at final concentrations of 900 nM and 100 nM respectively. Cycling conditions were: 50 °C, 5 min; 95 °C, 2 min; then 40 cycles of 95 °C, 3 s; 60 °C, 30 s, using a 7500 Fast real-time PCR machine (Applied Biosystems, UK).

### 2.8 Statistical analysis

Statistical analyses were performed using GraphPad Prism version 5.04 (GraphPad Software, Inc., USA).

## 3 Results and discussion

### 3.1 Vaccination regime optimisation

**Fig. 1:**
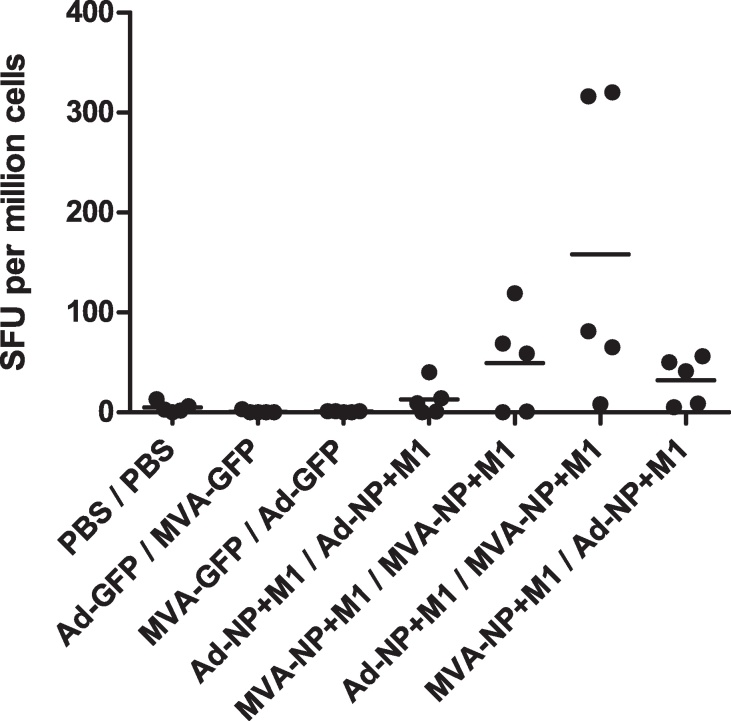
*Ex vivo* splenocyte IFN-γ ELISpot responses to NP + M1 peptides in Line 6_1_ chickens. Spot forming units (SFU) per million splenocytes in chickens sampled 10 days post-boost vaccination (*n* = 5 birds per group). Individual data points in all ELISpot results represent the sum of spot counts from 3 pools of 15–20-mer peptides overlapping by 10 amino acid residues, spanning the complete NP + M1 construct, with control peptide responses subtracted. Horizontal bars indicate mean values unless otherwise stated. Vaccination regimes are indicated on the *x* axis as prime/boost. Ad-NP + M1 prime followed by MVA-NP + M1 boost produced the highest mean antigen specific response. This response was significantly greater (one way ANOVA with Tukey post test) than Ad-NP + M1 prime/Ad-NP + M1 boost (*p* < 0.05), MVA-NP + M1 prime/Ad-NP + M1 boost (*p* < 0.05) and both irrelevant antigen (GFP) control regimes (*p* < 0.01).

Initial regime optimisation comprised *in ovo* prime vaccination at 17 days of incubation followed by boost at 4 weeks post hatch (wph) to determine the most immunogenic order of vector administration (Fig. 1). No adverse effects were observed following *in ovo* vaccination. Hatch rates were comparable to non-vaccinated embryos and the expected post hatch mortality rates were not correlated with vaccination. There were no observable changes in behaviour when measured by a sensitive scoring system approved for this purpose by the UK Home Office. Similarly, no adverse events were seen following IM boost post hatch. There were no effects at the site of injection other than a transient (1–2 days) inflammatory response. IFN-γ ELISpot with NP and M1 peptides was used to assess immunogenicity at 10 days post boost (dpb). Ad-NP + M1 prime followed by MVA-NP + M1 boost produced the highest mean antigen specific response. This response was significantly greater (one way ANOVA with Tukey post test) than Ad-NP + M1 prime/Ad-NP + M1 boost (*p* < 0.05), MVA-NP + M1 prime/Ad-NP + M1 boost (*p* < 0.05) and both irrelevant antigen (GFP) control regimes (*p* < 0.01). These results demonstrated that Ad prime followed by MVA boost was the most immunogenic regime and hence this was used for all subsequent regime optimisation. Our ability to detect antigen specific responses confirms that the transgene was expressed. Analysis of responses to individual peptide pools showed that responses to NP and M1 were similar (data not shown). Whilst we were unable to detect GFP expression by fluorescent microscopy we saw clear anti-GFP antibody responses, verifying expression of the control transgene (data not shown).

**Fig. 2:**
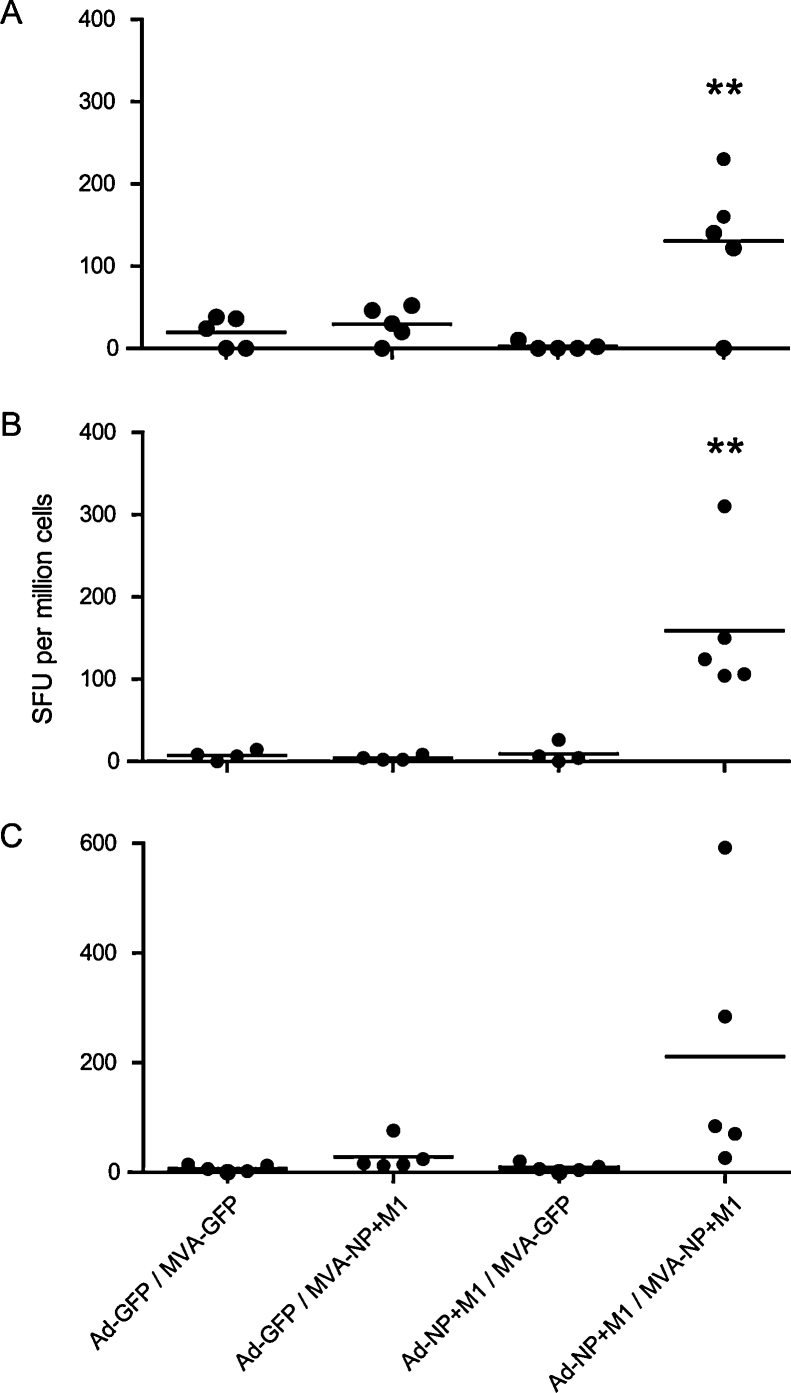
*Ex vivo* splenocyte IFN-γ ELISpot responses to NP + M1 peptides in out bred Light Sussex chickens (*n* = 5 birds per group). **Indicates statistically significant differences between heterologous prime-boost NP + M1 vaccinated birds and all control regimes (one way ANOVA with Tukey post test, *p* < 0.01). A and B represent 10 and 15 dpb responses respectively for birds primed *in ovo* at day 19 and boosted at 4 wph. C shows 10 dpb responses in birds primed at 2 weeks and boosted at 5 wph.

We next assessed immunogenicity of the Ad prime-MVA boost in two different age groups of outbred bird (Fig. 2). The first regime comprised *in ovo* Ad at day 19 of incubation and MVA boost at 4 wph. Splenocytes were used in IFN-γ ELISpots at 10 (Fig. 2A) and 15 (Fig. 2B) dpb. The second regime consisted of Ad prime at 2 wph and MVA boost at 5 wph, with ELISpots performed at 10 dpb (Fig. 2C). Ad-NP + M1 prime-MVA-NP + M1 boost induced the highest mean antigen specific response in the *in ovo* prime-4 wph boost groups (*p* < 0.01, figure 2A and B) and in the 2 wph prime and 5 wph boost group (*p* < 0.05 to all groups except Ad-GFP/MVA-NP + M1). These experiments clearly demonstrated the requirement for both prime and boost. While the 2–5 wph regime produced the highest mean SFU per million cell counts the results were more variable than in the younger cohort. For this reason and because *in ovo* vaccination is widely and increasingly used commercially we proceeded with the day 19 *in ovo* prime-4 wph boost regime.

### 3.2 Pre and post-challenge immune response

**Fig. 3:**
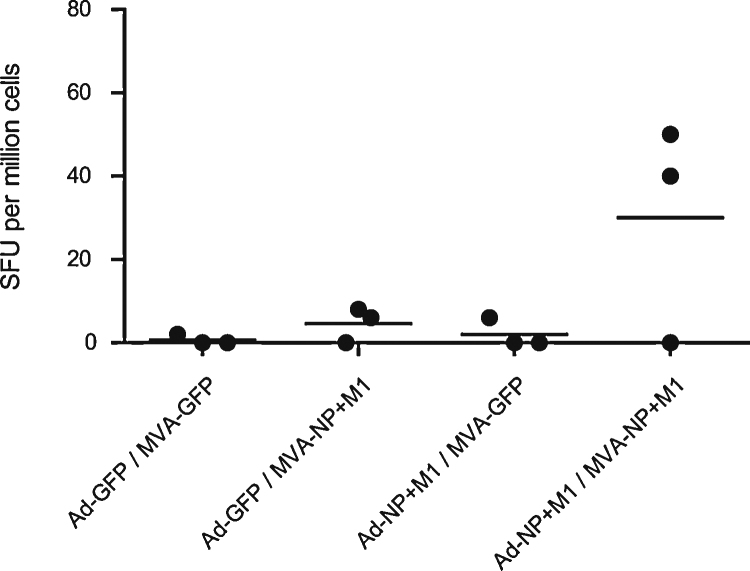
Pre challenge ELISpot responses to antigen. *Ex vivo* 10 dpb splenocyte IFN-γ ELISpot responses to NP + M1 peptides in inbred line 15I chickens (*n* = 3 birds per group).

Inbred line 15I chickens, known to be susceptible to the challenge virus, were vaccinated with the day 19 *in ovo* prime-4 wph boost regime. Splenocytes were used in IFN-γ ELISpots at 10 dpb (Fig. 3). Ad-NP + M1 prime-MVA-NP + M1 boost produced the highest mean antigen specific response although there is no statistically significant difference in this small study. The low SFU counts seen in the line 15I chickens compared with the out bred Light Sussex may reflect the restricted peptide binding repertoire of the line 15I major histocompatibility (MHC) haplotype [13]. One recent study was unable to predict any epitopes in NP or M1 for the line 15I haplotype [14]. The out bred Light Sussex express different MHC haplotypes for which peptide anchor residues are yet to be determined. Anti NP antibodies (we did not have an assay for M1) were detected in both the Ad-GFP/MVA-NP + M1 and the Ad-NP + M1/MVA-NP + M1 groups, however there was no significant difference between the antibody levels in these groups (data not shown).

**Fig. 4:**
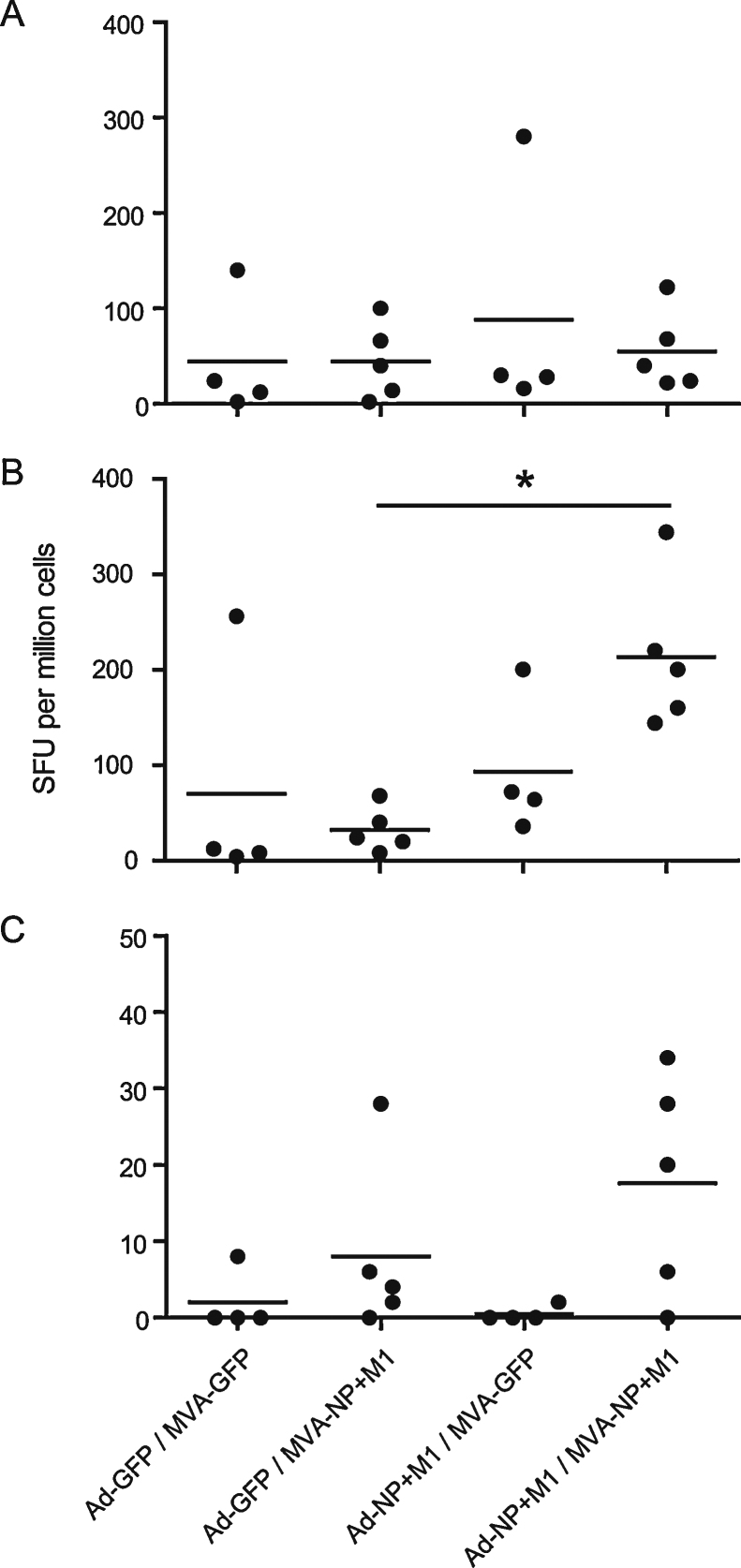
Post challenge ELISpot responses to antigen. *Ex vivo* 14 dpi splenocyte IFN-γ ELISpot responses to (A) NP + M1 peptides, (B) inactivated virus and (C) recombinant NP in inbred line 15I chickens (*n* = 4 birds per MVA-GFP boost group or 5 birds per MVA-NP + M1 boost group). *Indicates statistically significant difference between heterologous prime-boost NP + M1 vaccinated birds and the Ad-GFP prime-MVA-NP + M1 boost control regime (*p* < 0.05, one way ANOVA with Tukey post test).

At 14 days post challenge birds were euthanized and IFN-γ ELISpots performed (Fig. 4). We found no significant difference in the mean antigen specific response to peptide pools post challenge (Fig. 4A) although the mean responses were higher in all groups compared to 10 dpb (Fig. 3) indicating that as would be expected infection has boosted the response to NP and M1 peptides. Splenocytes were also cultured with inactivated challenge virus (Fig. 4B) to assess responses to a whole virus antigen preparation. The highest mean response was seen in the Ad-NP + M1 prime-MVA-NP + M1 boost group and was significant compared with the Ad-GFP prime-MVA-NP + M1 boost group (*p* < 0.05). IFN-γ ELISpot incorporating recombinant NP (Fig. 4C) produced very low mean responses with no significant differences seen between groups although the highest mean response was again in the Ad-NP + M1 prime-MVA-NP + M1 boost group. Interestingly analysis of the peptide ELISpot data revealed that an average of 42% of the IFN-γ-ELISpot response was attributable to NP, the majority of the response being to M1. This may explain the small changes in the response to recombinant NP and the relatively higher response to inactivated virus in the Ad-NP + M1 prime-MVA-NP + M1 boost group. Alternatively the inactivated virus data may reflect the relative abundance of M1 compared to NP during influenza infection.

### 3.3 Viral shedding

**Fig. 5:**
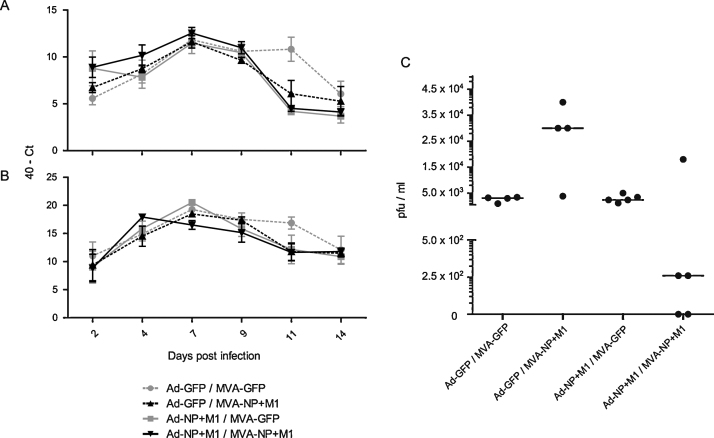
Time course of viral shedding and plaque assay at 7 days post infection. Viral shedding (mean + SEM) in (A) buccal and (B) cloacal swab samples measured by qRT-PCR for M1 and (C) by plaque assay (median indicated) at 7 dpi. Significant differences are discussed in the text. Pre-challenge swab samples were negative by qRT-PCR and plaque assay.

At 2 days post infection (dpi) all swabs were positive for M1 transcript by qRT-PCR. Pre-infection swab samples were negative (undetectable by qRT-PCR) as expected (data not shown). There were few significant differences in shedding detected by qRT-PCR. Relative amounts of transcript increased from 2 dpi to 7 dpi then generally decreased from 7 dpi to 14 dpi. At 7 dpi there were significant differences in 40 – *C*_t_ values between the Ad-GFP prime-MVA-GFP boost group and the Ad-NP + M1 prime-MVA-NP + M1 boost group and between the Ad-NP + M1 prime-MVA-GFP boost group and the Ad-NP + M1 prime-MVA-NP + M1 boost group (*p* < 0.05 and *p* < 0.01 respectively, one way ANOVA with Tukey post test). At 11 dpi the Ad-GFP prime-MVA-GFP boost group had significantly higher qRT-PCR scores in both the buccal and cloacal swabs than the other groups (*p* < 0.05 with Ad-GFP prime-MVA-NP + M1 boost group and *p* < 0.01 with the remaining groups, one way ANOVA with Tukey post test). Plaque assays at 4 dpi indicated that all birds were infected as virus was isolated from all buccal and cloacal swabs, with no significant differences between groups (data not shown). At 7 dpi buccal swabs were all negative by plaque assay (data not shown). Plaque assay of 7 dpi cloacal swabs (Fig. 5C) showed a substantial reduction in shedding in four out of five of the Ad-NP + M1 prime-MVA-NP + M1 boost group. This difference was not statistically significant, however two of the birds had ceased shedding and two birds were shedding virus at a level two logs lower than any of the birds in the control groups. These results suggest that this vaccination regime is effective in reducing cloacal shedding. In birds, transmission of influenza is *via* the faecal-oral route so this finding is noteworthy. The plaque assay score for the Ad-GFP prime-MVA-NP + M1 boost group was significantly different (*p* < 0.01, one way ANOVA with Tukey post test) compared with the other groups, although the biological significance of this result is unclear. At 14 dpi all plaque assay results were negative (data not shown).

Viral titres measured by qRT-PCR assays can be 2–3 logs higher than viral titres measured by plaque assay [15]. In addition, the well documented incidence of non infectious virion production following influenza virus challenge requires that infection based methods such as plaque assay should be used alongside PCR based methods [16]. Our present observation that plaque assays do not fully correlate with the results of qRT-PCR highlights the importance of performing assays of replicative virus.

Our results are consistent with previous studies showing that vaccination with Adenovirus expressing NP was immunogenic in chickens, inducing LPAI-specific effector memory T lymphocyte IFN-γ responses 1 week after boost vaccination [8]. Other research showed partial protection against mortality (though not clinical disease) after vaccination with NP DNA plasmids followed by challenge with highly pathogenic H5 and H7 influenza [17]. Our decision to include M1 as an antigen in this vaccine was supported by mammalian studies which identified M1 as a potentially cross protective antigen [3]. ELISpot responses to the M1 peptide pool were similar to ELISpot responses to both pools of NP peptides in terms of spot forming units, suggesting that anti-M1 responses may be as important as anti-NP responses in terms of T cells induced per unit of antigen. In addition recent studies in both mice and humans using MVA-NP + M1 [4,5], and preclinical data for Ad-NP + M1 (Mullarkey, submitted) support the inclusion of M1 as an antigen in these recombinant vaccines. A further benefit in chickens is that increasing the number of antigens in the vaccine increases the availability of epitopes where MHC haplotypes exhibit limited binding ability.

## 4 Conclusions

This is the first report of a challenge study evaluating the efficacy of NP + M1 as an antigen delivered by a prime-boost viral-vectored vaccination regime against influenza in chickens and describes for the first time successful *in ovo* vaccination of chicken embryos with recombinant MVA. The data reported herein show that vaccination regimes utilising MVA and Adenovirus incorporating the NP + M1 construct are safe for use *in ovo* and post hatch in prime/boost regimes, immunogenic and reduce the incidence of shedding of infectious influenza virus. Vaccination promoted a cellular immune response characterised by antigen specific production of IFN-γ. Following challenge with LPAI, two birds had ceased cloacal shedding and another two of five birds in the Ad-NP + M1 prime-MVA-NP + M1 boost group exhibited markedly reduced cloacal shedding of infectious influenza virus at 7 dpi as determined by plaque assay. While sterile immunity is the ultimate goal of vaccination, our results demonstrate for the first time the potential protective efficacy of NP + M1 construct prime-boost regimes in the control of virus shedding. Our work suggests that the inclusion of internal antigens in avian vaccines is advantageous, and provides the foundation for future work on the development and optimisation of more effective vaccination regimes utilising viral vectors to generate broadly heterosubtypic immunity either as sole vaccines or in combination with traditional or recombinant vaccines designed to elicit humoral immunity.
